# The clenched fist syndrome: case report of a clinical rarity of special interest for psychiatrists and hand surgeons

**DOI:** 10.1186/s12888-019-2348-4

**Published:** 2019-11-08

**Authors:** Abdulwares Meiwandi, Marios Papadakis

**Affiliations:** 1 Department of Plastic, Reconstructive, Aesthetic and Hand Surgery, Helios University Hospital Wuppertal, Witten-Herdecke University, Wuppertal, Germany; 2 Division of Surgery II, Witten-Herdecke University, Wuppertal, Germany

**Keywords:** Clenched-fist-syndrome, Psychoflexed hand, Hand contracture, Conversion disorder

## Abstract

**Background:**

The Clenched Fist Syndrome (CFS) is a type of a psychiatric disorder, in which the patients show flexion finger contractures. Although no organic etiology can be identified, the syndrome in most cases presents with pain and paradoxical stiffness.

**Case presentation:**

We, herein, report the case of a 52-year old woman with a 6-month history of progressive hand flexion contracture and intermittent numbness in the first 3 fingers, mimicking carpal tunnel syndrome. On examination, all digits, including the thumb, were held in a tight flexion at the metacarpal and interphalangeal joints. Passive digital extension was painless in all fingers. Physical examination did not reveal any joint tenderness, joint or tendon sheath swelling. X ray was performed and did not show any abnormalities. Neurological examination did not reveal any organic etiology.

**Conclusions:**

CFS is believed to be a conversion disorder, i.e. unconsciously motivated and produced, whereas others consider it a factitious disorder, i.e. unconsciously motivated but consciously produced. Surgical treatment is not indicated, as it can worsen the symptoms. The related literature is discussed. We conclude that CFS should always be considered in patients with unexplainable flexion hand contractures, especially in the presence of a positive psychiatric history.

## Background

Psychopathological hand disorders can present with various symptoms ranging from treatment-resistant ulcera (mostly self-inflicted) or swelling, abnormal sensory dysfunction and psychopathological hand dystonia [1, 2]. These disorders are not of somatic origin and organic pathologies should be excluded for the diagnosis to be established. Since these disorders are very rare, they are often overlooked, most times resulting in inadequate treatment and unnecessary progressive mutilating operations [3].

We herein report a patient with clenched fist syndrome (CFS). CFS is considered a conversion disorder [1, 4] and belongs to the group of psychopathological hand dystonias [3]. The condition is characterized by flexion contracture of several digits or in more severe cases of the entire hand. Patients with CFS often suffer from psychiatric comorbidities such as depression, schizophrenia or obsessive compulsive disorders. Treatment can be very difficult since patients generally lack insight into the psychiatric nature of their disease and most times are not willing to accept any additional psychiatric treatment [1, 2, 5–8].

## Case presentation

**Fig. 1:**
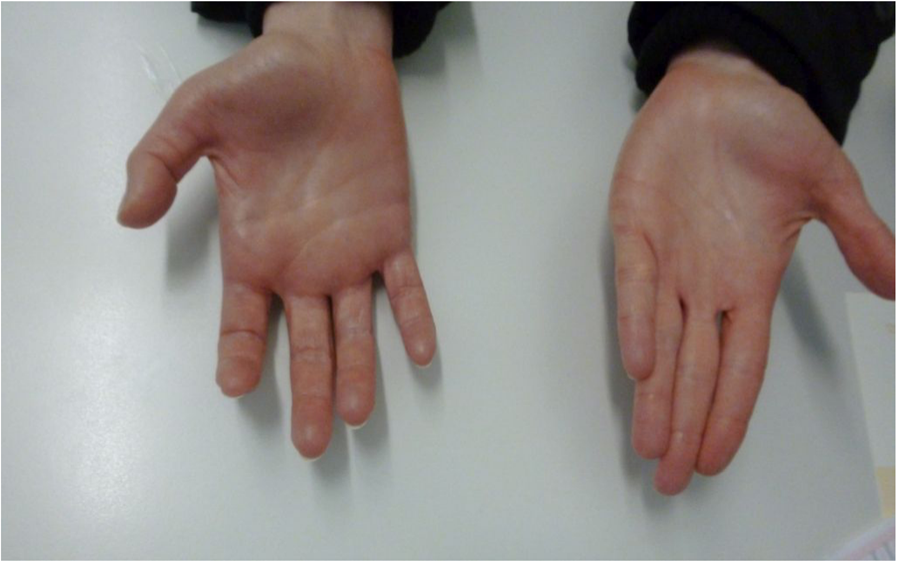
Clenched right hand with flexion contracture in metacarpal and interphalangeal joints

A 52-year-old woman presented to our department with a 6-month history of progressive hand flexion contracture. The patient had already consulted 19 hand surgeons without a diagnosis. She suffered from depression and obsessive-compulsive disorder, classified as mysophobia, but had not been receiving any medications. She reported diffuse pain in the hand without being able to recall any previous injury and intermittent numbness in the first three fingers of your hand. On physical examination all digits of the right hand, including the thumb, were held in a flexed position at the level of the proximal and distal interphalangeal joint (Fig. 1). Passive digital extension was painless in all fingers. Active extension was not possible. No signs of joint tenderness, joint or tendon sheath swelling were observed. Peripheral sensibility and blood perfusion was normal. Neurological examination did not reveal any organic etiology. Hand x-rays excluded bone injuries or degenerative changes, such as arthritic conditions. Electromyography of the brachial plexus and the peripheral nerves of the upper extremity did not show any pathologic conditions. MRI of the hand showed no abnormalities with relevance to CFS-symptoms. Based on these findings, the diagnosis of CFS was made. Although initially being susceptible to receiving treatment, Despite initially being susceptible in receiving treatment, the patient was treated with splint and intensive physiotherapy. She was already consulting a psychiatrist for the mysophobia and continued with biofeedback training and cognitive behavioral therapy (CBT). The hand was brought back to the neutral position after 1 month.

## Discussion and conclusion

CFS is a very rare clinical entity, with less than 40 cases reported. It is a psychiatric syndrome without clear and well-established etiology that can be assigned to the group of conversion disorders. Therefore, it is to be differentiated from factitious disorders or malingering [1, 2]. Patients suffering from CFS develop flexion contractures of the hand and fingers with potentially devastating outcomes if left untreated. The term clenched fist syndrome was first introduced in 1980 by Simmons et al. [3], who described a case series of five patients of different age with flexion contractures of the entire hand. Particular affected fingers were the ulnar three digits. In 1983, Frykman et al. used the term psycho-flexed hand to describe a case series of five patients, who, similar to the clenched fist syndrome, presented with flexion contractures [9]. In this case series, though, the dominant hand was involved. The patients were all middle-aged and none of them had the entire hand clenched. While there are differences between both reported patient groups, it is possibly the same entity that varies in symptoms.

In the past, many authors described CFS as a subgroup of SHAFT-syndrome, which is a factitious disorder, leading affected patients to seek polysurgery and manipulate medical staff in order to satisfy their psychological needs. These patients tend to be sad, hostile, anxious, frustrating and tenacious, so the acronym SHAFT was built [6]. Many recent reports indicate that CFS is a conversion disorder that is unconsciously motivated and unconsciously produced [1, 4, 7, 8]. Therefore, it should not be grouped with the factitious disorders, which are unconsciously motivated and consciously produced, or with malingering which is consciously motivated and consciously produced [1, 2, 10].

The DSM V criteria introduced the group of Functional Neurological Symptom Disorders as a subtype of conversion disorders, classified as F44.4 in the existing ICD 10 version. In the upcoming ICD 11 Classification these disorders are further categorized as Dissociative neurological symptom disorder, with movement disturbance (ICD Code: 6B60.8) [11]. In the literature these types of disorders are also often recognized as psychogenic movement disorders. This term emphasizes the psychogenic etiology [12]. Even though the terminology is not uniformly, the diagnostic criteria are very similar. In the upcoming ICD 11 “Dissociative neurological symptom disorders, with movement disturbance are characterized by symptoms such as chorea, myoclonus, tremor, dystonia, facial spasm, parkinsonism, or dyskinesia that are not consistent with a recognized disease of the nervous system, other mental and behavioural disorder, or other health condition and do not occur exclusively during another dissociative disorder” [11].

Patients with CFS present with a variety of symptoms ranging from simple flexion deformities to contraction in the entire hand. Even minor trauma seems to trigger the disease in most cases. Bilateral involvement is also possible, but not the rule. Most often the disorder involves both interphalangeal joints. Additional psychiatric comorbidities are almost always present in patients with CFS and a thoroughly psychiatric assessment should be performed in the first consultation [3, 7–9, 13, 14]. Our patient showed an unexplainable flexion contracture of all digits at the level of the proximal and distal interphalangeal (PIP and DIP) joints of the right hand resembling previously described cases with no prior trauma. In addition, the patient had a positive psychiatric history, as she was suffering from obsessive compulsion disorder and depression. Both disorders have been closely linked to the clenched fist syndrome in the literature [1, 5, 14]. Mysophobia has not been increasingly represented in the published cases though [2, 12, 14].

In the physical examination, the majority of patients show flexion contractures in the interphalangeal and metacarpophalangeal (MCP) joint, resulting in clenching the hand into a fist. The ulnar-sided fingers are most commonly affected. Active extension is not possible and trying to passively extend the fingers without anesthesia is too painful to accomplish. In advanced cases, the contractures become permanent due to changes in the soft tissue, joints and tendons. Macerations and infections due to palm hygiene problems also occur in these cases [3, 7, 8, 13]. In our patient, all fingers including the thumb were affected at the level of metacarpal and interphalangeal joints. There was no joint tenderness, joint or tendon sheath swelling. Passive digital extension was painlessly possible in all fingers.

Radiography, laboratory tests, MRI and electromyography are in most cases normal, but comprise the standard diagnostic tools used to rule out any organic etiology before CFS diagnosis can be made. All of these tests were carried out in our patient and showed no pathological results. Differential diagnosis includes rheumatologic diseases, Dupuytren contracture, camptodactyly, complex regional pain syndrome, central neurological and peripheral nerve diseases. The fact that in our case all 19 hand surgeons being consulted, missed the diagnosis is not uncommon for CFS, since it is a relatively rare disease and most physicians are not aware of psychopathological hand disorders [1–3, 5, 6, 8, 9].

The treatment of CFS consists of unclenching the hand under anesthesia, intensive physiotherapy and psychotherapy (e.g. CBT, biofeedback, hypnosis) [1, 2, 8, 15]. In prolonged cases, it is sometimes indicated to relieve the contractures surgically. Recent reports show promising results in patients that underwent successful psychotherapy. Surgery should only be performed in these patients and not on psychiatric unstable patients. Fixed chronic contractures that cannot be resolved through conservative treatment or intensive physiotherapy represent an indication for surgery. These contractures develop when the hand is held in a fixed clenched position for a long period of time [7, 8].

Our patient was educated about the psychopathological nature of her disorder and received splint and intensive physiotherapy. The goal of CBT was to convince the patient that the pathology of her hand could not be attributed to a clear organic etiology and to develop coping strategies [1, 15]. In our opinion, both therapy modalities (physiotherapy and psychotherapy) contributed to the improvement, as the patient could not see the necessity of physiotherapy without psychotherapy. The hand was brought back to the neutral position after 1 month.

Prognosis is described as rather poor in literature since most of the patients tend not to comprehend or neglect the graveness of their illness. Thus, treatment is not completed and drop outs are fairly common. The success of therapy correlates closely to the results of psychotherapy [1, 3, 4, 8].

We conclude that CFS should always be considered in patients with unexplainable flexion hand contractures, especially in the presence of a positive psychiatric history.

## Data Availability

Data sharing is not applicable to this article as no datasets were generated or analysed during the current study.

## Abbreviations

CBT: Cognitive Behavioural Therapy
CFS: Clenched-Fist-Syndrome
DIP: Distal InterPhalangeal Joint
DSM V: Diagnostic and Statistical Manual of Mental Disorders V
ICD: International Classification of Diseases
MCP: MetacarpoPhalangeal Joint
MRI: Magnetic Resonance Imaging
PIP: Proximal Interphalangeal Joint
SHAFT: sad, hostile, anxious, frustrating and tenacious
